# Effect of exogenous amino acids application on the biochemical, antioxidant, and nutritional value of some leafy cabbage cultivars

**DOI:** 10.1038/s41598-022-21273-6

**Published:** 2022-10-21

**Authors:** Maryam Haghighi, Amir Barzegar Sadeghabad, Reza Abolghasemi

**Affiliations:** Department of Horticulture, College of Agriculture, Isfahan University of Technology, Isfahan, Iran

**Keywords:** Physiology, Plant sciences

## Abstract

Using organic fertilizer as part of plant nutrition for decreasing using chemical fertilizer and increasing plants’ nutritional value is scientists’ concern. Treatments were three concentrations of a mixture of 16 different AAs (amino acid) (0, 150 and 300 mg/L), sprayed every 7 days for 2 months on 4 leafy cabbages. Results showed 300 mg/L AAs increased anthocyanin, flavonoids, phenol, protein and proline. The SOD, POX and APX rose upon AAs usage. The application of AA significantly increased the total chlorophyll, proline, carotenoid, anthocyanin, phenol, protein and flavonoids compared to control plants. The levels of glucosinolate were increased especially in the treatment of 300 mg/L of AAs and glucobrassicin and gluconapin, both of these together represent more than 50% of the total glucosinolate contents. The highest levels of phenolic and flavonoids mostly belonged to quercetin and catechin. Total AAs and total non-essential AAs showed the highest amounts in all treatments in leaves. AAs with different concentrations by foliar application in “Ka- scotch” variety were effective in growth, physiological parameters such as plant height and shoot dry weight, while AA changes were effective in most of the biochemical and nutritional traits of “Ka-red” variety. Conclusively, the glucosinolate, phenolic and flavonoid contents and AAs varied between four cabbage cultivars. Exogenous AAs application at 300 mg/L could be recommended for cabbage cultivation to improve growth, biochemical traits, productivity and nutritional value.

## Introduction

Cabbage (*Brassica oleracea* var. *capitata*) is a leafy vegetable belonging to the *Cruciferae* family that is consumed annually^1^. Due to its high nutritional value, including vitamins, antioxidants, protein, carbohydrates, amino acids (AAs) and mineral nutrition^1^, cabbage is a beneficial vegetable for humans. The growth, productivity and nutritional value of this important plant are, however, affected and constrained by environmental conditions^2^. External application of essential AAs could protect plants from environmental conditions and increase their nutritional value^3^.

A foliar spray of macro and micronutrients is highly effective for plant growth and yield^4^. Nitrogen is the substance required in the greatest quantity for plant growth. Consequently, it plays a crucial role in the metabolism and vegetative growth of plants, and it is one of the principal agents limiting crop efficiency^4^. Due to its carcinogenic properties, excessive use and storage of this nutrient element in vegetables raises a significant threat to human health^4^. Due to their raw consumption, most vegetables, such as cabbage, serve as a source of nitrate storage in the human body and contribute significantly to the daily nitrate intake^5^. Therefore, it is essential to find a way to control the accumulation of nitrate in vegetables or to find a way to reduce this element’s concentration in them^5^. Urea is one of the most common sources of nitrogen for foliar application; it increases leaf photosynthesis and leaf urease activity^5^. Recent research indicates that replacing nitrate nutrition with exogenous AAs reduces this element’s accumulation in leafy vegetables^6^. As AAs are the final product of nitrate uptake and reduction in plants can increase the concentration of AAs in plant tissues, it can inhibit nitrate uptake and accumulation by plant root cells^6^. Researchers found that foliar application of AAs increased the activity of nitrite reductase enzymes and decreased nitrate accumulation in lettuce^6^. Soybean nitrate uptake and accumulation were inhibited when nitrate in nutrient solution was replaced with a mixture of essential AAs^1^. In addition, exogenous AAs have a positive effect on nitrate accumulation reduction in cabbage^1^, Pak-choi (*Brassica chinensis* L.)^7^, and radish^8^. According to numerous reports, one of the nutritional concerns is excessive nitrate accumulation in vegetables, so it needs to be found a valuable and cost-effective nitrogen source^1^.

Normal situations recognize amino acids as stimulants of quantitative and qualitative roles in plant growth^4^. These compounds are crucial for the synthesis of hormones and secondary metabolites^4^. AAs are substances with a role in metabolic processes that enhance plant performance. These compounds can also play coenzyme roles^5^; amino acids are constituents of proteins and are utilized in numerous essential plant growth pathways.

Some research has focused on the foliar application of AAs because they are readily absorbed when applied externally^3^. The beneficial effects of AAs on numerous plant species have been investigated. Among these effects are the enhancement of nutrient, water and photosynthesis absorption by various vegetables^1^, the promotion of flower production and the maintenance of fruit production^9^, and the improvement of the physiological parameters and biochemical composition of cabbages^3^. In addition, the use of AAs has significantly raised the vegetative growth of lettuce^6^ in hydroponic conditions and radish (*Raphanus sativus*) in soil fields^8^.

In soil cultivation, foliar application of essential amino acids or a hydroponic nutrient solution supplement is far more beneficial than irrigation^10^. In this regard, it has been reported that foliar AA application increases plant growth, yield, and quality in potato (*Solanum tuberosum*)^5^, garlic (*Allium sativum*)^11^ and bean (*Phaseolus vulgaris*)^12^. In another study, the application of AA mixtures to broccoli seedlings (*Brassica oleracea* var. *Italica*) resulted in a significant increase in the vegetative growth of the shoots and roots^1^. In a similarly, Mobini et al.^5^ reported that leaf application with three distinct AAs improved plant growth, yield, and bulb quality.

In addition, it has been stated that the use of AAs increases the nutritional value of certain agricultural products^3^. By increasing the concentration of AAs, proteins and other amino acids increase the nutritional value of plants^3^. Researchers report that the proportion of individual amino acids found in cabbage leaves varies significantly. For instance, an AA spray reduced the accumulation of nitrate in cabbage, bulb and radish crops^2,5,8^. According to researchers, foliar application of AAs increases the protein content of beans^12,13^. Yunsheng et al.^13^ reported that foliar application of glutamine at concentrations between 25 and 100 mg/L enhanced shoot growth, total AAs, protein, total phenol in the shoot and crop production.

There have been studies on the effects of external application of AAs on a variety of plants, but the effects on cabbages remain unclear. We hypothesized that external application of AAs could increase the yield, biochemical properties, and nutritional value of cabbage by enhancing growth and antioxidant enzyme. To test this hypothesis, we examined the impact of external application of AAs on the growth, biochemical, and nutritional parameters of leafy cabbages. Consequently, the purpose of this study was to: (1) determine the effect of AAs on growth, biochemical, and nutritional parameters; and (2) compare the effects of external AAs on four leafy cabbages. No studies have yet compared the effects of AAs on different cabbage cultivars. Therefore, it is prudent to comprehend the effects of external AAs on different cultivars of cabbage grown under identical conditions and their interactions. In light of this, this study examined the effect of leaf application of various AA mixtures on the biochemical, enzymatic, and nutritional characteristics of several cabbage cultivars. This study’s findings may be useful for future research. They can help develop the foliar application of essential AAs to improve the biochemical properties and nutritional value of agricultural products.

## Results

### The effect of different cultivar and AA on the growth parameters of 4 leafy cabbages

The result of analysis showed that the main effect (Table 1) and interactions effect (Table 2) of AAs and cultivars on all growth characteristics of cabbages such as; plant height, number of leaves per plant, chlorophyll index, shoot fresh and dry weight, root fresh and dry weight were significantly different (P ≤ 0.05). ANOVA result of growth parameters presented in Table S1.

**Table 1:** The main effect of AA and cultivar on some growth characteristics of cabbages.

	Height (cm)	Number of leaves per plant	Chlorophyll (SPAD value)	Shoot fresh weight (g)	Shoot dry weight (g)	Root fresh weight (g)	Root dry weight (g)
Collard	19.27a	26.22a	17.18a	154.6b	23.9c	21.3a	2.1a
Ka-red	14.33c	23.00b	12.01c	141.6c	20.7d	20.5b	1.8 b
Ka-smooth	18.56b	22.33b	17.94a	155.4b	25.5b	20.6b	2.1a
Ka-scotch	18.93ab	26.55a	13.94b	168.5a	27.4a	21.0ab	2.1a
Control	16.11c	22.04c	12.82b	12.9c	16.7c	12.1c	1.4c
AA1	17.81b	24.91b	15.85a	164.3b	26.7b	24.3b	2.3b
AA2	19.40a	26.62a	17.14a	177.9a	29.7a	26.1a	2.5a

*Control* control treatment, *AA1* 150 mg/L of amino aci, *AA2* 150 and 300 mg/L of amino acid.

Within a column in each treatment means followed by the same letter are not significantly different at P < 0.05 according to the least significant difference test.

**Table 2:** Interaction effect of cabbage cultivar and amino acid application on growth parameters.

		Height (cm)	Number of leaves per plant	Chlorophyll (SPAD value)	Shoot fresh weight (g)	Shoot dry weight (g)	Root fresh weight (g)	Root dry weight (g)
Collard	C	27.36ef	141.6ef	12.57de	119.6e	15.9hi	12.5e	1.4e
	AA1	29.41bc	168.3bc	18.38ab	160.5 b	26.6d	24.9bc	2.3bc
	AA2	31.05a	176.6b	20.60a	183.5a	29.3c	26.4a	2.7a
Ka-red	C	22.78i	110.0g	10.69e	115.1e	14.7i	11.8e	1.0f
	AA1	24.30h	131.6f	12.18de	149.2c	22.7f	24.5cd	2.3c
	AA2	25.91g	148.3de	13.17cde	160.6b	24.8e	25.1bc	2.3bc
Ka-smooth	C	26.60fg	93.3h	15.72bc	121.3e	17.0h	11.6e	1.7d
	AA1	28.55cd	135.0ef	19.10a	164.8b	27.8d	23.4d	2.3bc
	AA2	30.53a	141.6ef	19.0a	180.2ab	31.6b	26.7a	2.5ab
Ka-scotch	C	27.70de	136.6ef	12.30de	135.6d	19.2g	12.6e	1.7d
	AA1	29.00c	161.6cd	13.74cd	182.7ab	29.9c	24.3cd	2.3bc
	AA2	30.11ab	198.3a	15.80bc	187.2a	33.1a	26.0ab	2.5abc

*Control* control treatment, *AA1* 150 mg/L of amino acid, *AA2* 150 and 300 mg/L of amino acid.

According to the least significant difference test, a column in each treatment and means followed by the same letter is not significantly different at P < 0.05.

The main effect of most growth parameters such as plant height, number of leaves per plant and shoot fresh and dry weight, root fresh and dry weight showed that the AA2 has a more significant effect than control (Table 1). One cultivars, “Ka-scotch” improved shoot fresh and dry weight more than the others significantly, and the lowest of these parameters was observed in “Ka-red” (Table 1).

The interaction effect of AA and cultivars showed that the there is a significant trend with increasing the concentration of AA used in all leafy cabbages in plant height, dry and fresh weight of shoot (Table 2). The lowest plant height, dry weight of shoot and root were observed in control treatment in “Ka-red” cultivar. Also, the highest number of leaves and shoot dry weight was observed in “Ka-scotch” cultivar using 300 mg/L of AAs. In this regard, 300 mg/L treatment in most cultivars was superior in fresh and dry weight of root (Table 2). The lowest number of leaves were detected in “Ka-smooth” at control (Table 2).

### The effect of cultivar and AA on the biochemical parameters of 4 leafy cabbages

The main effects of AA and cultivars on the biochemical traits of leafy cabbages are presented in Table 3. Cultivar and AAs showed significant differences at 0.05 levelin biochemical parameters of cabbage such as total chlorophyll, proline, carotenoid, anthocyanin, phenol, protein, flavonoids (Table 3). ANOVA result of biochemical parameters presented in Table S2. The AA application significantly increased the total chlorophyll, proline, carotenoid, anthocyanin, phenol, protein and flavonoids compared to control plants. So, the highest number of biochemical traits was observed in 300 mg/L (Table 3). The study of the antioxidant enzymes such as APX, SOD and POX showed that the activities of these enzymes were significantly affected by AAs. The main effects showed that APX and POX were increased by applying the AAs; thus, these enzymes’ lowest activity was observed in the control treatment (Table 4). The highest SOD was observed in 300 mg/L of AAs. In contrast, CAT was not affected by the amino acid concentration (Table 4).

**Table 3:** The main effect of AA and cultivar on some biochemical characteristics of cabbages.

	Total chlorophyll (mg/100 g FW)	Proline (µmol/g FW)	Carotenoids (mg/100 g FW)	Anthocyanin (mg/100 g FW)	Total phenol content (mg/100 g FW)	Protein (mg/g FW)	Flavonoids (mg/g FW)
Collard	14.31c	3.20b	0.12b	0.80b	33.15bc	0.18c	0.40c
Ka-red	15.32ab	2.93c	0.11c	1.39a	34.15ab	0.18a	0.45a
Ka-smooth	14.98bc	3.57a	0.13a	0.70c	35.43a	0.17c	0.40c
Ka-scotch	15.92a	3.71a	0.13a	0.733c	32.52c	0.18b	0.41b
Control	12.72c	2.72c	0.11c	0.73c	29.02c	0.17c	0.36c
AA1	15.39b	3.41b	0.13b	0.89b	34.36b	0.18b	0.42b
AA2	17.29a	3.94a	0.14a	1.09a	38.05a	0.19a	0.46a

*Control* control treatment, *AA1* 150 mg/L of amino acid, *AA2* 150 and 300 mg/L of amino acid.

Within a column in each treatment means followed by the same letter are not significantly different at P < 0.05 according to the least significant difference test.

**Table 4:** The main effect of AA and cultivar on some antioxidant and glucosinolate contents of cabbages.

	APX (unit mg/protein)	SOD (unit mg/protein)	POX (unit mg/protein)	CAT (unit mg/protein)	Glucoraphanin µM/g DW	Progoitrin µM/g DW	Gluconapin µM/g DW	Glucobrassicin µM/g DW	Gluconasturtiin µM/g DW
Collard	0.74b	0.55b	0.78b	0.40b	15.31d	7.07d	13.83d	25.505d	2.11 c
Ka-red	1.12a	0.56b	1.08a	0.24c	23.22b	13.31b	23.04b	37.66b	3.53a
Ka-smooth	0.68b	0.61b	0.70b	0.54a	19.69c	10.25c	18.77c	32.15c	2.91b
Ka-scotch	0.96a	0.91a	1.05a	0.40b	26.31a	14.96a	28.62a	41.00a	3.91a
Control	0.60b	0.56b	0.72b	0.38a	20.51c	10.58c	18.60c	32.48c	2.82b
AA1	1.08a	0.65b	1.04a	0.41a	21.01b	11.41b	21.57b	34.10b	2.94b
AA2	0.95a	0.77a	0.96a	0.39a	21.88a	12.20a	23.02a	35.66a	3.58a

*Control* control treatment, *AA1* 150 mg/L of amino acid, *AA2* 150 and 300 mg/L of amino acid, *APX* ascorbate peroxidase, *SOD* superoxide dismutase, *POX* peroxidase, *CAT* catalase.

Within a column in each treatment means followed by the same letter are not significantly different at P < 0.05 according to the least significant difference test.

**Figure 1:**
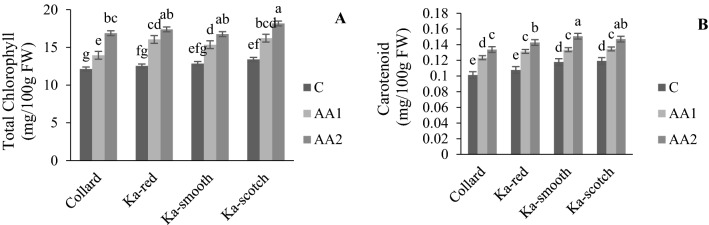
Interaction effect of cabbage cultivar and AA application on total chlorophyll (**A**) and carotenoid (**B**). *C* control treatment, *AA1* 150 mg/L of amino acid, *AA2* 150 and 300 mg/L of amino acid.

**Figure 2:**
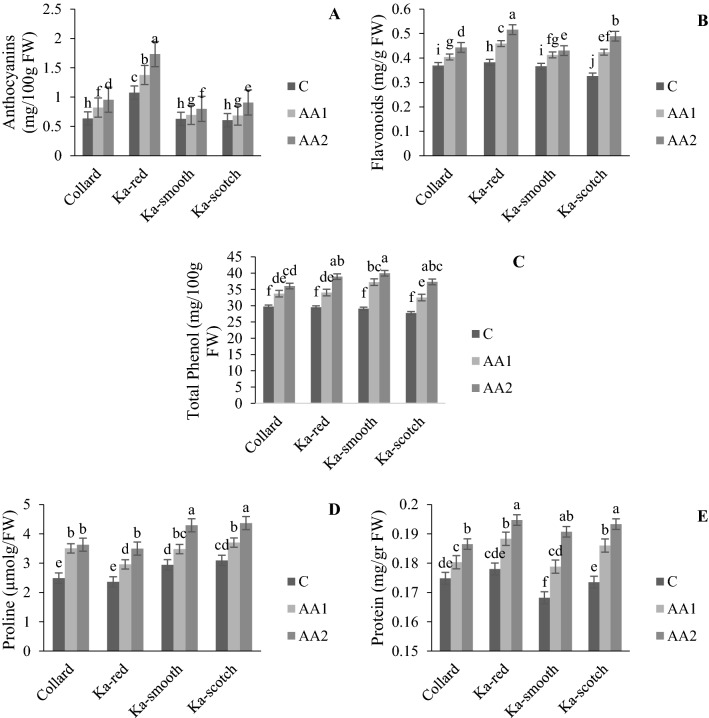
Interaction effect of cabbage cultivar and AA application on anthocyanins (**A**), flavonoids (**B**), phenol (**C**), proline (**D**) and protein (**E**). *C* control treatment, *AA1* 150 mg/L of amino acid, *AA2* 150 and 300 mg/L of amino acid.

**Figure 3:**
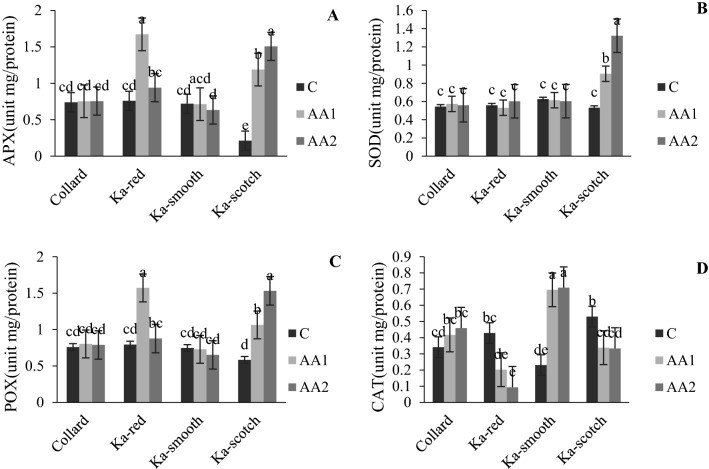
Interaction effect of cabbage cultivar and AA application on antioxidant enzyme activities. *APX* ascorbate peroxidase, *SOD* superoxide dismutase, *POX* peroxidase, *CAT* catalase, *C* control treatment, *AA1* 150 mg/L of amino acid, *AA2* 150 and 300 mg/L of amino acid.

The AA and cultivar interaction effect showed that photosynthesis pigments (chlorophyll and carotenoid) of the cabbage were significantly increased by applying AAs (Fig. 1A,B). Photosynthetic pigments in each cultivar increased when the AA concentration increased (Fig. 1A,B). It was also observed that the levels of anthocyanin, flavonoids, phenol, proline and protein increased with the application of higher concentrations of AAs compare to control treatment in all of the cultivars (Fig. 2A–E). Increasing the AAs concentration significantly increased the proline and protein content to its highest content with 300 mg/L in all of the cabbage cultivars while the lowest proline and protein content were observed in the control treatment (Fig. 2D,E). The interaction effect of cultivar and AAs application on antioxidant enzymes (APX, SOD and POX) showed significant difference between the cultivars and the amount of used AAs, and specially in “Ka-scotch” a clear trend of three antioxidant enzymes was observed with increasing AA concentration (Fig. 3A–C). This means that the changes in the interaction effects of cabbage cultivar and AA application on these three enzymes were similar. The use of AAs did not show a clear trend in the activity of CAT with increasing AAs concentration, an upward trend was observed in cultivars “Collard” and “Ka-smooth”, while in “Ka-red” and “Ka-scotch” a downward trend was observed (Fig. 3D). In the present study, the activity of antioxidant enzymes was observed with external AA spray in an optimum environment. The “Ka-scotch” cultivar showed a significant increase in the activity of APX, SOD and POX (Fig. 3A–C).

### The effect of cultivar and AA on the nutritional value (glucosinolate, phenolic, flavonoids and amino acid) of 4 leafy cabbages

The study of an extract of four leafy cabbages allows the detection of glucosinolate, phenolic, flavonoids and amino acid contents and the quantification of the main compounds by HPLC. Five glucosinolates such as glucoraphanin, progoitrin, gluconapin, glucobrassicin and gluconasturtiin were detected in this study. ANOVA result of nutritional value presented in Supplementary Table S3. Glucosinolate showed a remarkable main effect between AA concentration and cabbages (Table 4). The lowest glucosinolate contents were observed in “Collard” and the highest content of these compounds was observed in “Ka-scotch” cultivar (Table 4). The AA treatment increased all of the glucosinolate contents. The glucosinolate contents increased by 300 mg/L with increasing the AA concentration, and the lowest contents were observed in the control treatment (Table 4). The predominant glucosinolates were glucobrassicin and glucoraphanin. The mean value of these glucosinolates was 32.48 and 20.51 µM/g DW (Table 4). Interaction effects of AA and cultivars on glucoraphanin, progoitrin, gluconapin, glucobrassicin and gluconasturtiin were significant (Table 5). The glucosinolate content in most of the cultivars increased when they were treated with AAs compare to control treatment (Table 5). For glucosinolate contents, there were notable differences among varieties. Thus, this would be effective for the future study focused on cultivars of high or low glucosinolate contents. The highest glucoraphanin, progoitrin, gluconapin and glucobrassicinwere observed in “Ka-scotch” cultivar with external using of AAs (Table 5). Concerning glucobrassicin and gluconapin, both these together represent more than 50% of the total glucosinolate contents (Table 5). Gluconasturtiin did not show statistically significant changes in all study varieties by increasing AAs’ amount (Table 5).

**Table 5:** Interaction effect of cabbage cultivar and AA application on glucosinolate content µM/g DW.

		Glucoraphanin	Progoitrin	Gluconapin	Glucobrassicin	Gluconasturtiin
Collard	Control	16.07g	6.70g	12.29i	24.12i	1.87e
	AA1	14.96h	6.74g	13.78h	25.00i	1.73e
	AA2	14.91h	7.78f	15.41g	27.38h	2.73cde
Ka-red	Control	22.59c	12.51c	18.55e	36.09d	3.53abc
	AA1	22.84c	13.20bc	24.35c	37.54c	3.05bcd
	AA2	24.23b	14.21b	26.23b	39.35b	4.02ab
Ka-smooth	Control	18.46f	9.23e	16.83f	30.64g	2.44de
	AA1	19.67e	10.31d	18.83e	32.25f	3.11bcd
	AA2	20.94d	11.22d	20.64d	33.56e	3.18bcd
Ka-scotch	Control	24.91b	13.89b	26.73b	39.06b	3.46abc
	AA1	26.58a	15.38a	29.32a	41.60a	3.86ab
	AA2	27.45a	15.61a	29.80a	42.34a	4.42a

*Control* control treatment, *AA1* 150 mg/L of amino acid, *AA2* 150 and 300 mg/L of amino acid.

According to the least significant difference test, a column in each treatment and means followed by the same letter is not significantly different at P < 0.05.

The HPLC analysis allowed the quantification of 15 phenolics and three flavonoid contents identified in 4 leafy cabbages. They are 14 phenolic contents: gallic acid, chlorogenic acid, vanillic, caffeic acid, rutin, p-coumaric acid, catechin, ferulic, benzoic, acacetin, pyrogallol, genistein, cinnamic acid, luteolin and flavonoids contents: quercetin, kaempferol and myricetin. For phenolic and flavonoid contents, the main effect analysis showed significant differences (Table 6). ANOVA result of phenolics and flavonoid contents presented in Table S4,S5. Significant differences were detected between phenolic and flavonoid contents among cultivars and AA. For leafy cabbages, there were notable differences among phenolic contents for all of the varieties. In contrast, only “Ka-scotch” cultivar did not show significant changes in phenolic contents, and with increasing the concentration of AA, no changes in phenolic contents were observed (Table 6). In all cultivars in control catechin showed the highest among phenolic compounds i.e. the highest amount of catechin (12.53 mg/100 g DW) was observed in “Ka-red” control treatment (Table 6).

**Table 6:** Interaction effect of cabbage cultivar and AA application on phenolic acids and flavonoid parameters.

		Collard			Ka-red			Ka-smooth			Ka-scotch		
	(mg/100 g DW)	Control	AA1	AA2	Control	AA1	AA2	Control	AA1	AA2	Control	AA1	AA2
Phenolic compound	Gallic acid	3.9d	3.07b	3.67c	3.1b	2.807c	2.58c	1.406b	1.289bc	1.199bc	1.869d	1.707b	1.624a
	Chlorogenic acid	1.59de	0.43b	2.29c	0.79c	2.173c	1.854d	0.487b	1.037c	0.911c	1.477d	1.051b	1.444a
	Vanillic	nd	0.38b	0.32c	0.77c	1.824c	1.841d	0.48b	0.899c	0.905c	nd	1.045b	1.345a
	Caffeic acid	nd	0.70b	0.39c	0.8c	1.956c	1.929d	0.491b	0.951c	0.94c	nd	1.054b	1.382a
	Rutin	5.36d	5.76b	13.48b	4.49b	3.208c	3.318b	1.958b	1.449b	1.492a	4.655c	2.102b	1.738a
	P-coumaric acid	1.67de	1.97b	10.72b	1.47c	2.195c	2.277c	0.758b	1.046bc	1.079b	3.871cd	1.244b	1.450a
	Catechin	11.34a	15.92a	12.34ab	12.53a	7.597a	6.109a	9.128a	3.193a	2.601a	7.171a	7.225a	2.984a
	Ferulic	7.82c	1.45b	1.3c	5.19b	3.884bc	2.135c	2.236b	1.717b	1.022bc	1.196d	2.3b	1.93a
	Benzoic	14.4b	1.80b	2.15c	6.34b	5.691b	2.231c	2.693b	2.435ab	1.06c	1.437d	2.627b	2.443a
	Acacetin	8.06c	3.32b	1.78c	4.55b	3.95b	2.648c	1.982b	1.743b	1.226b	1.332d	2.119b	1.948a
	Pyrogallol	6.12c	6.22a	5.12c	4.12b	3.417c	6.191a	1.811b	1.532b	2.634a	2.281cd	1.997b	1.797a
	Genistein	2.57d	2.57b	2.22c	0.27c	2.442c	2.442c	Nd	1.144bc	1.144b	1.457d	0.903b	1.52a
	Cinnamic acid	nd	0.38b	1.17c	0.31c	1.94c	1.841d	0.297b	0.944c	0.905c	Nd	0.915b	1.377a
	Luteolin	9.65c	5.82b	38.56a	2.95bc	4.387b	3.335b	1.346b	1.917b	1.499a	1.777d	1.664b	2.072a
Flavonoid	Quercetin	16.33aB	15.98aB	12.0aB	15.56aA	11.71aB	6.67aC	7.94aA	4.82aB	2.82aC	7.07aA	6.38aB	4.15aC
	Kaempferol	1.88bAB	2.05bA	0.33bB	3.23bA	2.25bA	2.29bA	1.45bA	1.06bA	1.08abA	0.92bB	1.74bA	1.46bAB
	Myricetin	0.76bA	0.661bAB	0.56bB	0.82bA	1.94bA	1.91bA	0.49bB	0.94bA	0.93bA	0.98bA	1.05bA	1.37bA

*Nd* not detected, *Control* control treatment, *AA1* 150 mg/L of amino acid, *AA2* 150 and 300 mg/L of amino acid.

According to the least significant difference test, a column in each treatment and means followed by the same letter is not significantly different at P < 0.05. Upper letter showed significant effect of AA application on flavonoid in each row.

In this study, the content of flavonoids including quercetin, kaempferol, and myricetin showed a significant difference with AA (Table 6). The table showed that flavonoid contents decreased or did not change significantly in all studied cultivars (Table 6). As can be seen in that table, quercetin had the highest content in all treatments and varieties studied. The highest amount of quercetin (16.33 mg/100 g DW) was observed in the control treatment of “Collard” cultivar. Also, with AAs in different concentrations, “Collard” cultivar showed the highest amount of quercetin in this study (Table 6). In contrast, myricetin had the lowest contents among all studied cultivars and AA concentrations (Table 6).

As the ANOVA and main effects are presented in Table S6, both of cultivars and concentration of AAs applied on total AAs of cabbage were significantly different (P ≤ 0.05). Significant changes in total AAs were observed with increasing the concentration of exogenous AAs (Table 7).

**Table 7:** Interaction effect of cabbage cultivar and AA application on different total AAs.

	Collard			Ka-red			Ka-smooth			Ka-scotch		
	Control	AA1	AA2	Control	AA1	AA2	Control	AA1	AA2	Control	AA1	AA2
Total sulfur AA	2.53b	3.25b	3.39b	4.05b	5.17b	5.39b	2.02b	2.29b	2.34b	2.51b	3.23b	3.37b
Total aromatic AA	5.01b	6.10b	6.66b	7.95b	9.65b	10.53b	3.48b	3.88b	4.09b	4.97b	6.05b	6.61b
Total essential AA	5.17b	6.45b	6.92b	8.21b	10.19b	10.93b	3.54b	4.01b	4.18b	5.14b	6.40b	6.87b
Total non-essential AA	24.47a	25.65a	25.96a	38.54a	40.37a	40.86a	13.92a	14.36a	14.47a	24.27a	25.44a	25.755a
Total AAs	40.21a	45.27a	46.76a	63.57a	71.42a	73.75a	25.17a	27.04a	27.59a	39.90a	44.91a	46.39a

*Control* control treatment, *AA1* 150 mg/L of amino acid, *AA2* 150 and 300 mg/L of amino acid.

According to the least significant difference test, a row in each treatment and means followed by the same letter is not significantly different at P < 0.05.

The interaction effects of cultivars and AA application in leaves are reported in Table 7. All AA compounds (total sulfur AAs, total aromatic AAs, total essential AAs, total non-essential AAs, and total AAs) were affected by AAs’ exogenous application (Table 7). The leaves’ total non-essential AAs have the highest amount among other compounds, and total sulfur AAs yielded the lowest when AAs were applied (Table 7). The amount of total AAs in all cabbage cultivars in this study ranged from 73.75 mg/100 g FW belonging to “Ka-red” cultivar in AA2 treatment and 25.17 mg/100 g FW belonging to “Ka-smooth” cultivar in the control treatment (Table 7).

## Discussion

### The effect of cultivar and AA on the growth parameters of 4 leafy cabbages

Under favorable environmental conditions, amino acids are beneficial biological compounds that play a significant role in plant growth and development. The application of external AAs has a number of direct and indirect effects on plants, including physiological activities and photosynthesis traits that promote plant growth, development and yield^1^. Consequently, a significant increase in plant height, number of leaves, chlorophyll, fresh and dry weight of shoots and roots was observed as AA concentration increased in the present study. Under drought stress, Haghighi et al.^2^ found that the application of AAs spray (150 mg/L) enhanced the growth and vegetative characteristics of cabbage. Accordingly, the application of 300 mg/L of AAs increased the fresh weight of shoots by 38% and the dry weight of shoots by 72% in the “Ka-scotch” cultivar in the present study. The results of this study mirrored those of Shekari and Javanmardi^1^, who sprayed broccoli plants with 200 mg/L of AAs. In the study conducted by Barker and Pilbeam^14^, it was found that a 750 mg/L increase in exogenous AAs increased the amount of radish growth factors.

### The effect of cultivar and AA on the biochemical parameters of 4 leafy cabbages

AAs have beneficial effects on plant growth and biochemical characteristics. It has been reported that the increase in chlorophyll and carotenoid content in treated plants may be attributable to the presence of AAs, which increase photosynthesis pigments^13^. AAs are essential compounds that play a crucial role in the synthesis of plant compounds such as proteins, amines, secondary metabolites, enzymes, phenols and flavonoids, which regulate various plant processes^13,15^. In this study with AAs sprayed on the plant, an increase in the amount of these compounds was observed in all cultivars, confirming previous research. In support of our findings, researchers have demonstrated that AAs spraying has positive effects on phenolic and flavonoid compounds^5^.

According to reports, leafy AA spraying increases antioxidant enzymes under environmental stress^15^. Similarly, in this study, this increase in proline and protein content may be attributable to the direct effect of the exogenous AAs used on cabbage^16^. As a source of nitrogen, AAs are essential for the production of plant proteins, and the use of AAs at various concentrations increased the protein content of beans^12^. According to Haghighi et al.^2^, the use of AAs increases the amount of proline and protein as a useful osmolyte in plants, which is related to the concentration of AAs. Previous studies have shown that spraying AAs on cabage^2^ and *Aloe vera*^10^ leaves significantly increases antioxidant activities such as SOD, POX and APX. Similarly, the concentration of SOD, POX, and APX was increased by applying and increasing AAs under normal conditions, particularly in the “Ka-scotch” cultivar of cabbage. It has been reported that antioxidant enzymes play the primary role in controlling free radicals; as a result, their stimulation can increase the plant’s resistance to environmental stress and delay senescence^15^. Our findings supported the findings of other researchers^17^. The hypothesis is that AA can enhance antioxidant capacity. This increase was observed in both enzyme antioxidants and non-enzyme antioxidants, such as flavonoids and phenols^1,3,18^. It increases the nutritional value of cabbage.

### The effect of cultivar and AA on the nutritional value (glucosinolate, phenolic, flavonoids and amino acid) parameters of 4 leafy cabbages

Glucosinolates are sulfur-containing metabolites in cabbage plants, and the use of external AAs^19^ affects their accumulation. Exogenous AA increased glucosinolate levels in “Ka-scotch” more than in other cultivars; glucoraphanin, progoitrin, gluconapin, glucobrassicin and gluconasturtiin levels rose by 10.1%, 12.3%, 11.4%, 8.7% and 27.7%, respectively. In this regard, it appears that increasing glucosinolate can improve the nutritional value of this vegetable. As demonstrated in this study, foliar application of AAs can increase this nutritional quality in cabbage cultivars, particularly in “Ka-Scotch”. The external application of the amino acid methionine increases glucosinolate synthesis in Arabidopsis and broccoli, according to our findings^20,21^. In support of our findings, it has been reported that AAs play the primary role in glucosinolate synthesis in plants^22^. According to the findings of researchers, the range of phenolic and flavonoid content varies by species, cultivar and AAs content^23^. The principal phenolic compounds in cabbages are flavonols, such as quercetin, kaempferol, caffeic, p-coumaric and sinapic acids, and they are used for chemical plant protection^24^. Exogenous AAs altered cabbage phenolic compounds. In cabbage, cultivars differed in phenolic content, whereas cauliflower^24^ had the highest phenolic acid content. In the present study, a relatively high amount of catechin was found in the control treatment of “Ka-red” confirming this issue. Flavonoids are present in the epidermis of cabbage leaves and play a crucial role in absorbing ultraviolet light, protecting against insects and physical and environmental stresses, and inhibiting reactive oxygen species^25^. Important flavonoids in cabbages have been identified as quercetin, kaempferol and Myricetin^24^. In the present study, all cultivars with exogenous AAs had the highest quercetin levels. In general, the phenolic and flavonoid content of cabbage in our study was comparable to previous research^24^. The use of AAs increases the nutritional value of cabbage and radish^1,8^. The following compounds have been reported in other cabbages, such as Chinese cabbage^17^. By increasing the concentration of AAs, proteins and other amino acids increase the nutritional value of plants. Researchers report that the proportion of individual amino acids found in cabbage leaves varies significantly^3^. In our study, this was confirmed by the fact that the greatest variation in amino acid content was between total sulfur AA and total non-essential AA.

**Figure 4:**
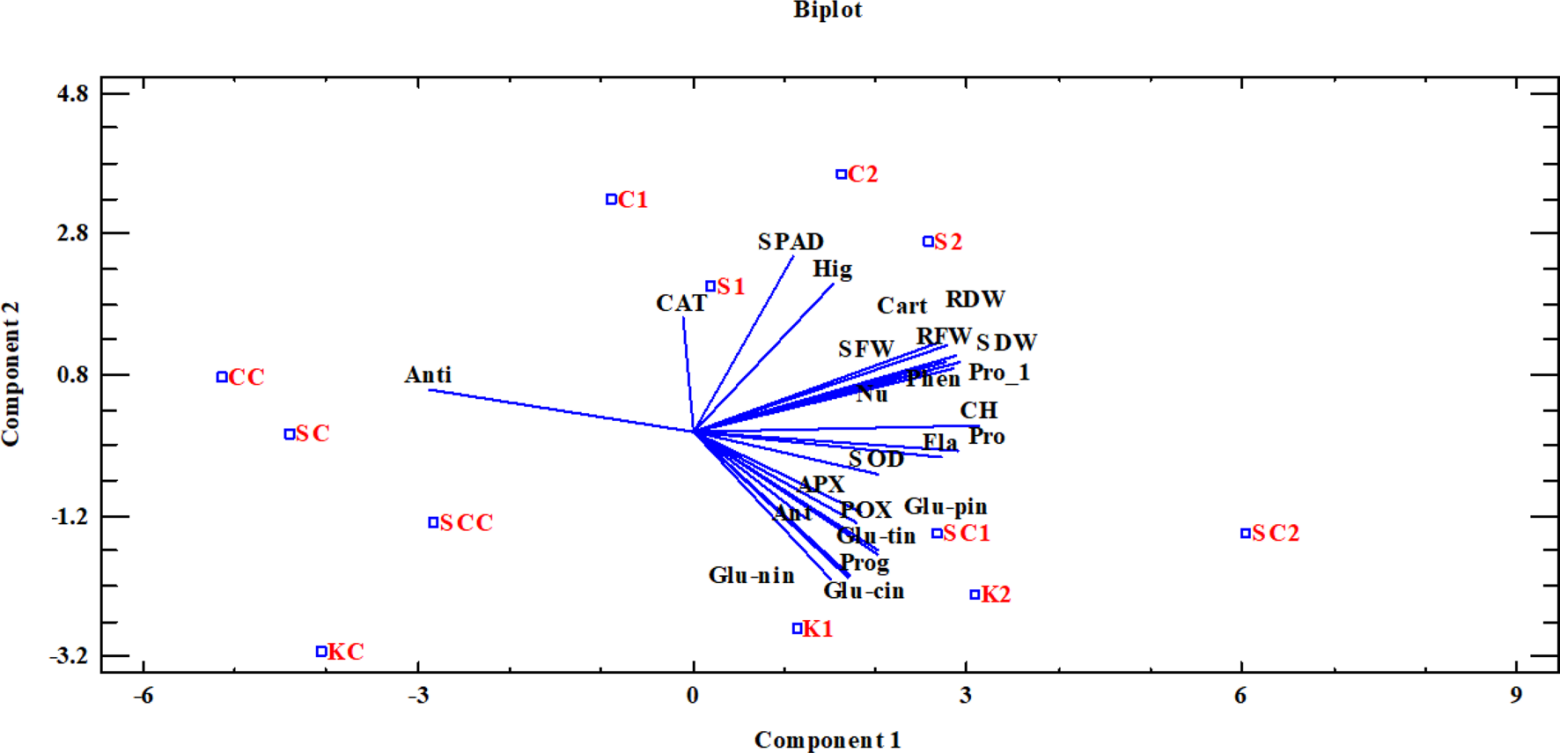
PCA analysis of all the evaluated parameters in cabbage cultivars. Collard & 0 mg/L amino acid (CC), Collard & 150 mg/L amino acid (C1), Collard & 300 mg/L amino acid (C2), Ka-red & 0 mg/L amino acid (KC), Ka-red & 150 mg/L amino acid (K1), Ka-red & 300 mg/L amino acid (K2), Ka-smooth & 0 mg/L amino acid (SC), Ka-smooth & 150 mg/L amino acid (S1), Ka-smooth & 300 mg/L amino acid (S2), Ka-scotch & 0 mg/L amino acid (SCC), Ka-scotch & 150 mg/L amino acid (SC1), Ka-scotch & 300 mg/L amino acid (SC2), Height (hig), Number of leaves per plant(Nu), Chlorophyll (SPAD), Shoot fresh weight (SFW), Shoot dry weight (SDW), Root fresh weight (RFW), Root dry weight (RDW), Total Chlorophyll (CH), Proline (Pro-1), Carotenoid (Cart), Anthocyanin (Ant), Phenol (Phen), Protein (Pro), Flavonoids (Fla), Antioxidant (Anti), Catalase (CAT), Peroxidase (POX), Ascorbate peroxidase (APX), Superoxide dismutase (SOD), Glucoraphanin (Glu-nin), Progoitrin (Prog), Gluconapin (Glu-pin), Glucobrassicin (Glu-cin), Gluconasturtiin (Glu-tin).

PCA analysis revealed that all studied factors, including growth, biochemical, and nutritional factors, were aligned with one another, and with the exception of antioxidant activity, which was distinct from the others. The PCA figure confirmed the other findings of this study, so that in all varieties, the control treatments were grouped together and only their antioxidant activity was comparable, whereas the application of AA as a spray significantly altered and increased the measurement factors. In general, this analysis reveals that the application of different concentrations of AAs by foliar application in “Collard” and “Ka-smooth” cultivars were effective for growth and physiological parameters such as chlorophyll (SPAD) and plant height, whereas AA changes were effective for biochemical and nutritional traits in “Ka-red” and “Ka-scotch” cultivars (Fig. 4).

**Figure 5:**
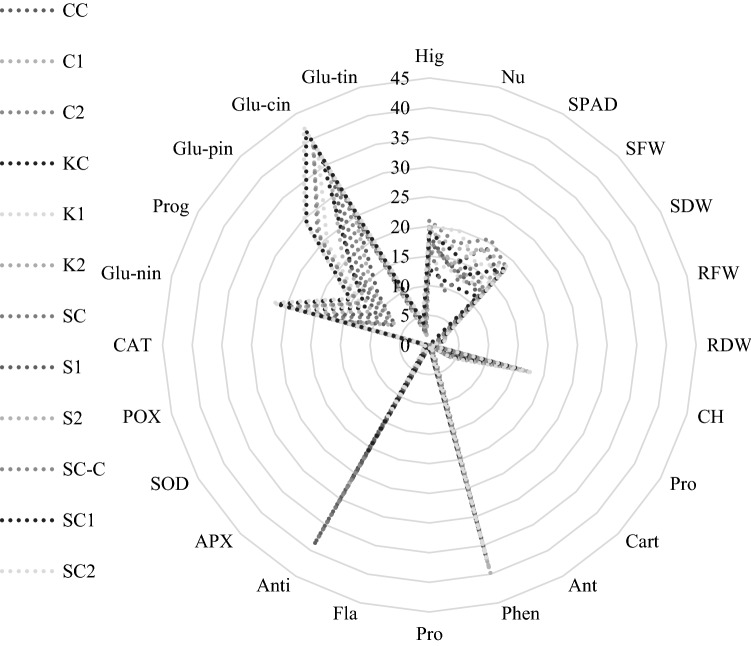
The interaction effect of AA and cabbage cultivars on all vegetative and nutritional traits. Collard & 0 mg/L amino acid (CC), Collard & 150 mg/L amino acid (C1), Collard & 300 mg/L amino acid (C2), Ka-red & 0 mg/L amino acid (KC), Ka-red & 150 mg/L amino acid (K1), Ka-red & 300 mg/L amino acid (K2), Ka-smooth & 0 mg/L amino acid (SC), Ka-smooth & 150 mg/L amino acid (S1), Ka-smooth & 300 mg/L amino acid (S2), Ka-scotch & 0 mg/L amino acid (SCC), Ka-scotch & 150 mg/L amino acid (SC1), Ka-scotch & 300 mg/L amino acid (SC2), Proline (Pro), Carotenoid (Cart), Anthocyanin (Ant), Phenol (Phen), Protein (Pro), Flavonoids (Fla), Antioxidant (Anti), Catalase (CAT), Peroxidase (POX), Ascorbate Peroxidase (APX), Superoxide Dismutase (SOD), Glucoraphanin (Glu-nin), Progoitrin (Prog), Gluconapin (Glu-pin), Glucobrassicin (Glu-cin), Gluconasturtiin (Glu-tin). Height (Hig), Number of leaves per plant (Nu), Chlorophyll (SPAD), Shoot fresh weight (SFW), Shoot dry weight (SDW), Root fresh weight (RFW), Root dry weight (RDW), Total Chlorophyll (CH).

The spider diagram revealed that among the studied parameters, improved chlorophyll (SPAD), shoot fresh weight, total chlorophyll, phenol, antioxidant activity and plant height, chlorophyll concentration, glucoraphanin and glucobrassicin were the most distinguishing characteristics between all treatments and varieties. Therefore, based on this figure and the changes observed when spraying foreign AAs on leafy cabbages in optimal environmental conditions, it can be stated that the use of AAs has been beneficial to important growth, physiological, biochemical, and nutritional traits of cabbages, and based on the findings of this study, the use of AAs is recommended in optimal environmental conditions to improve the growth and nutritional value of cabbages (Fig. 5).

## Material and methods

### Plant material and growing conditions

In this study, four leafy cabbage cultivars (*Brassica oleracea* var. smooth german kale (Ka-Smooth), *Brassica oleracea* var. scarlet kale (Ka-Scarlet), *Brassica oleracea* var. Dwarf Blue Curled Kale (Ka-red), *Brassica oleracea* var. collard (Vates) (Collard) were used from Seed Savers Exchange Company, Iowa, USA. We compiled all the relevant institutional, national, and international guidelines and legislation for the cultivation of plants were followed. The investigation was planned based on a complete randomized design (CRD) with three replicates consisting of 21 plants in each replication at the Isfahan University of Technology, Isfahan, Iran, in March 2019. The minimum and maximum temperatures were 9.2 and 20.5 °C, respectively. The cabbage seeds were cultivated at 2/1/1 cocopeat/perlite/peat moss ratio. Cabbage seedlings with 7–8 real leaves were transferred to 5 kg pots. The AAs used in this experiment were in powder form, consisting of a mixture of 16 AA compounds: (arginine, cysteine, glycine, phenylalanine, valine, threonine, tryptophan, methionine, leucine, glutamin https://en.wikipedia.org/wiki/Isoleucine, lysine and histidine, alanine, aspartic acid, asparagine, glutamic acid). The amount of each amino acid in 100 g of powder mixture is given in Table 8. The authors used this AAs in previous studies^2^. A solution with three concentrations 0, 150 and 300 mg/L was prepared and sprayed every 7 days for 2 months. Fertilizing and irrigation were conducted as cultivation recommendations in the region.

**Table 8:** Amino acid profile of 16 different AAs.

Amino acids	Amount mg/100 g powder
Arginine	43 mg
Cysteine	43 mg
Glycine	39 mg
Phenylalanine	50 mg
Valine	70 mg
Threonine	75 mg
Tryptophan	35 mg
Methionine	60 mg
Leucine	80 mg
Glutamin	75 mg
Isoleucine	80 mg
Lysine	85 mg
Histidine	60 mg
Alanine	84 mg
Aspartic acid	85 mg
Asparagine	55 mg
Glutamic acid	65 mg

### Growth and physiological parameters

The plants were harvested at the time of the economic harvest, and some leaves from each replication were immediately placed in frozen liquid nitrogen for further investigation.

The fresh weight of cabbage at harvest time was measured using an accurate scale (0.01 balances) and their dry weight after drying in the oven at 70 °C.

### Chlorophyll index

The chlorophyll index of mature plant leaves was used by the chlorophyll meter (SPAD-502 plus, Japan). For this goal, three readings were carried out from each plant on three separate leaves (a total of nine readings per replicate), and then the average was registered^26^.

### Chlorophyll and carotenoids contents (mg/100 g FW)

Some fresh leaf tissue (5.0 g) was mixed with 80% acetone, then filtered and balanced to 10 mL, the absorbance reading at 476, 646 and 663 nm using the spectrophotometer^17^ (UV 160A- Shimadzu Corp., Kyoto, Japan). The formula was used from Pérez-Grajales et al.^27^.

Chlorophyll a = (19.3 × A663—0.86 × A646) Volume/100 Weight.

Chlorophyll b = (19.3 × A646—3.6 × A663) Volume/100 Weight.

Total chlorophyll = Chlorophyll a + Chlorophyll b.

Carotenoids = 100(A476)—3.27(mg/g Chl. a)—104 (mg/g Chl. b)/227.

### Biochemical parameters

#### Protein content (mg/g FW)

Total protein content was set on the method of Bradford^28^. The protein standard was Bovine serum albumin. Fresh texture (1.0 g) was mixed with Na-Phosphate buffer (4 mL, pH = 7.2) and then centrifuged (4 °C). Added 5 ml of dye reagent to 100 μl extract and mixed well. At the same time, prepared a set of standards containing 5, 10, 20, 30, 40, 50 and 100 μl of Bovine Serum Albumin (BSA 2.0 mg/ml stock in extraction buffer) in separate tubes. Added extraction buffer to each tube to bring the volume to 100 μl. These tubes also added 5 ml of dye reagent and mixed well by overtaxing. After 5 min, read the absorbance at 595 nm against a reagent blank (100 μl of extraction buffer with 5 ml of dye reagent).

#### Proline (mg/g FW)

The ninhydrin test^29^ characterizes the concentrations of Proline. Sulfosalicylic acid (3%) was used to homogenize leaf samples at 4 °C. Then, the resulting solution was incubated and centrifuged (5000 rpm for 20 min). The supernatant was blended with ninhydrin (2.5%), phosphoric acid (60%; V/V), and glacial acetic acid (100%; 1 mL). The absorbance was recorded at 518 nm.

#### Anthocyanin content (mg/100 g FW)

The spectrometric method by Rapisarda et al.^30^ was used to determine the anthocyanin. Fresh leaves (1.0 g) were crushed with 20 mL alcohol (60%; pH = 3.0) and heated the samples on the hot water for 2 h; after cooling the samples certain volume of sample solution was used for reading at 535 nm.

#### Total phenol content (mg/100 g FW)

The total phenolic content of samples was evaluated by the method of Folin–Ciocalteu^22^. Folin–Ciocalteu reagent was diluted with distilled water (ten times). The soluble samples of kale extract (20 μL) were blended with diluted Folin-Ciocalteu reagent (1 mL), sodium bicarbonate solution (7.5%; 1 mL), and distilled water (1 mL). The resulting mixture was kept at room temperature for 15 min. The absorbance was recorded at 730 nm. The calibration curve of the samples was equivalent to gallic acid equivalents (GAE). The total phenolic content was reported as mg gallic acid equivalents of 100 g fresh leaves^31^.

#### Total flavonoid content (mg/g FW)

Flavonoid content was measured by the protocol of Yazdizadeh^31^, with minor changes. 0.2 mL of leaves extract with 0.8 mL distilled water was blended with AlCl_3_ (2%; 1 mL) in methanol solution (5% acetic acid in methanol). This combination stays at room temperature (10 min) and the absorbance was recorded at 430 nm. The sample blank was without reactant. Quercetin sample was applied as the standard for the calibration curve. The total flavonoid content of the samples was reported as mg quercetin equivalents (QE) of mg/g fresh weight of leaves.

#### Identification and quantification of phenolic and flavonoid components (mg/100 g FW)

For the preparation of leaves extracts in this study, 100 mg of leaves were blended with HPLC-grade methanol (80%; 10 mL) and shacked (8 h; 110 rpm; 25 °C). Then the extracts samples were filtered with the acrodisk of nylon (0.22 μm). The HPLC analysis was done by Lin & Harnly^32^ method. Symmetry C18 column (Waters Crop., Milford, MA, USA) in size of 4.6 mm × 250 mm (5 μm) was used as the stationary phase and the mobile phase consisted of a combination of formic acid (0.1%) and acetonitrile which was injected into the column (injection rate was 0.8 mL/min). All of the standards of phenolic components including gallic acid, chlorogenic acid, vanillic, caffeic acid, rutin, p-coumaric acid, catechin, ferulic, benzoic, acacetin, pyrogallol, genistein, cinnamic acid and luteolin and flavonoids including quercetin, kaempferol, and myricetin were prepared from Company of Sigma. These compounds were dissolved using HPLC grade methanol and, after that, with 20 μL injection volume, were injected into the analytical column. Chromatograms were received at a wavelength range of 200–400 nm. The amount of flavonoids and phenolic compounds in cabbage leaves extracts was evaluated based on their retention times and peak area with their standards. Finally, they were reported as mg per 100 g of dried leaves weight. One model of chromatogram kale leaves injection is shown in Supplementary Fig. S1.

#### Identification and quantification of amino acids (mg/100 g DW)

Leaf samples were collected from the same place in different treatments, and the homogenized and random sample of pulp was used for amino acid measurement at the end of the experiment. The samples were hydrolyzed using HCl (6 M) in an ampoule including 10 mg phenol at 110 °C for 24 h, to protect tyrosine. After acid hydrolysis, the samples were diluted to 100 mL using a citrate buffer having sulfur-containing AAs. After pre-hydrolysis oxidation with performic acids, cysteine, and methionine were specified^33^. The identification and quantification of the individual AAs of the leaves were carried out by HPLC (Unicam Crystal 200 HPLC system, England). An MD-1510 diode-array detector set at 263 nm (λmax) was fitted into a Unicam Crystal 200 HPLC system (England). By using a 7125 valve (Rheodyne, Cotati, CA), a 20-μL loop was injected into the Purospher RP-18 column of the samples. By using the acetate buffer (50 mM; pH 4.2) as the eluent A and acetonitrile as the eluent B, with the column at a flow rate of 1.0 ml/min, at 25 °C. The level of AAs was indicated in 100 g of edible leaves.

#### Antioxidant enzyme activities (unit mg/protein)

Catalase (CAT) activity was measured spectrophotometrically (UV 160A- Shimadzu Corp., Kyoto, Japan) by the decrease of absorbance of H_2_O_2_ at 240 nm as described. This combination contained potassium phosphate buffer (50 mM; pH = 7.0), H_2_O_2_ (10 mM), and an enzyme extract (200 µL). The amount of CAT required to decompose by H_2_O_2_ (1.0 μM) and defined as one unit of CAT activity/min^25^. Peroxidase (POX) activity was determined using the increase in absorbance at 470 nm of guaiacol oxidation for 3 min. The reaction mixture consisted of phosphate buffer (25 mM; pH = 7.0), 0.05% guaiacol, 1.0 mM H_2_O_2_ and 0.1 mL of extract. POX activity indicates the amount of enzyme catalysis and the oxidation of 1.0 μM of guaiacol/1 min. Ascorbate peroxidase (APX) activity was measured according to Khoshbakht et al.^23^ by controlling ascorbate oxidation at 290 nm. The sample blending consisted of 50 mM potassium phosphate buffer (pH = 7.0), H_2_O_2_ (0.1 mM), ascorbic acid (0.5 mM), and enzyme extract (200 µL). The activity was reported as unit/mg of protein^10,23^. Total superoxide dismutase (SOD) activity was determined by controlling the inhibition of photochemical reduction of nitroblue tetrazolium by the method of Beauchamp in Khoshbakht et al.^23^. The 3 mL of reaction blending consisted of potassium phosphate buffer (50 mM; pH = 7.8), methionine (13 mM), nitroblue tetrazolium (75 µM), riboflavin (2 µM), EDTA (0.1 mM), and enzyme extract (100 µL). The reaction blending was lighted up for 15 min SOD activity was specified as the enzyme required for 50% inhibition of NBT reduction recorded at 560 nm^23^.

#### Glucosinolate content (µM/g DW)

For determining the glucosinolate content, 10 g of fresh tissue of leaves was weighted, kept at 120 °C for 2 h smashed to a powder using a mixer machine, transferred to a vial, and blended with 8 mL of 70% V/V methanol/water (of HPLC grade). 2 mL of the extracted liquids was purified (0.45 μm pore size) into vials. HPLC system was used (Unicam, crystal 200), consisting of bin pump G1312B, thermostatic column compartment G1316A, and diode array detector DAD-VL G1315D. The HPLC column was a 150 mm × 2 mm (2.8 μm particle size) Pursuit XRs Ultra 2.8 diphenyl column. The mobile phase was formic acid (0.005%) with an acetonitrile/water gradient over 75 min at a flow rate of 0.5 mL/min. The gradient profile increased from 5 to 75% linearly in 45 min followed by a return to 5% and 30 min isocratic to re-equilibrate. The absorbance was recorded by a spectrophotometer (UV 160A- Shimadzu Corp., Kyoto, Japan) at 229 nm^20^.

### Statistical analysis

ANOVA was analyzed with Statistix 8 (Tallahassee FL, USA), and the least significant difference (LSD) was used for means comparison at P ≤ 0.05. Biplot analysis used Statgraphics (Centurion XVII) for PCA analysis.

## Conclusion

This study demonstrated that foliar spraying with essential AAs significantly increased cabbage’s growth, biochemical and antioxidant characteristics, as well as its nutritional value. The use of AAs mixtures with higher concentrations was found to significantly improve the majority of the investigated parameters. In this regard, the highest AA concentration of 300 mg/L had the greatest effect on cabbage’s growth, antioxidant capacity, and nutritional value. Consequently, this nutritional factor could be used to increase the nutritional value and antioxidants in cabbage cultivation, including anthocyanin, flavonoids, phenol, protein, proline and a few essential AAs components. In addition, AAs increase antioxidant enzyme activity, which can prepare cabbage for potential environmental stress during plant development and boost the plant’s resistance. This study also provided valuable information regarding the nutritional value of cabbage, including its glucosinolate, phenolic acid, flavonoid, and total AA content in four cultivars. Due to the effects of AAs on cabbage’s growth, antioxidant, biochemical, and nutritional properties, it can be used as a foliar spray to help cabbage grow and improve farming quality.

## Supplementary Information

Supplementary Information.

## Data Availability

The data that support the findings of this study are available on request from the corresponding author.
