# Supplementary material for: Effect of exogenous amino acids application on the biochemical, antioxidant, and nutritional value of some leafy cabbage cultivars
Source: Sci Rep. 2022 Oct 21;12:17720. doi: 10.1038/s41598-022-21273-6 (PMC9587009; doi:10.1038/s41598-022-21273-6)
Supplement: Supplementary file 1 — Supplementary Information. [file 41598_2022_21273_MOESM1_ESM.docx]

**Supplementary**

**Table S1.** Analysis of variance of AA and cultivars on some growth characteristics of cabbages.

| Source | df | Height | Number of leaves per plant | Chlorophyll index | Shoot fresh weight | Shoot dry weight | Root fresh weight | Root dry weight |
| --- | --- | --- | --- | --- | --- | --- | --- | --- |
| Block | 2 | 0.3693 | 1.056 | 9.924 | 0.226 | 0.01427 | 0.00882 | 0.00033 |
| Cultivars | 3 | 96.4559^*^ | 84.796^*^ | 139.12^*^ | 21.695^*^ | 1.42324^*^ | 0.02626^*^ | 0.00406^*^ |
| AA | 2 | 65.0376^*^ | 128.764^*^ | 118.043^*^ | 196783^*^ | 11.1287^*^ | 13.7473^*^ | 0.07377^*^ |
| AA × Cultivars | 6 | 0.7052^*^ | 4.116^*^ | 11.695^*^ | 1.552^*^ | 0.0646^*^ | 0.0219^*^ | 0.00193^*^ |
| Total error | 58 | 1.5788 | 3.435 | 29.344 | 1.465 | 0.379 | 0.0290 | 0.00083 |
| CV |  | 7.07 | 12.76 | 35.47 | 7.81 | 7.96 | 8.17 | 13.62 |

ns: not significant, ** significant at 1% and * significant at 5%.

**Table S2.** Analysis of variance of AA and cultivars on some biochemical characteristics of cabbages.

| Source | df | Total chlorophyll | Proline | Carotenoids | Anthocyanin | Total phenol content | Protein | Flavonoids |
| --- | --- | --- | --- | --- | --- | --- | --- | --- |
| Block | 2 | 0.547 | 0.17176 | 0.00010 | 0.00102 | 15.021 | 0.0000027 | 0.00061 |
| Cultivars | 3 | 8.099^**^ | 2.25092^**^ | 0.00084^**^ | 1.91537^**^ | 29^**^ | 0.0000225^**^ | 0.00958^**^ |
| AA | 2 | 126.499^**^ | 9.03849^**^ | 0.00624^**^ | 0.79206^**^ | 494.865^**^ | 0.00188^**^ | 0.07165^**^ |
| AA × Cultivars | 6 | 1.248^**^ | 0.14635^**^ | 0.00004^**^ | 0.06804^**^ | 9.078^**^ | 0.0000377^**^ | 0.00353^**^ |
| Total error | 58 | 1.769 | 0.24548 | 0.00011 | 0.01111 | 8.737 | 0.00000467 | 0.00226 |
| CV |  | 8.78 | 14.75 | 8.11 | 11.58 | 8.74 | 3.74 | 11.35 |

ns: not significant, ** significant at 1% and * significant at 5%.

**Table S3.** Analysis of variance of AA and cultivars on some antioxidant and glucosinolate contents of cabbages.

| Source | df | APX | SOD | POX | CAT | Glucoraphanin | Progoitrin | Gluconapin | Glucobrassicin | Gluconasturtiin |
| --- | --- | --- | --- | --- | --- | --- | --- | --- | --- | --- |
| Block | 2 | 0.01461 | 0.00981 | 0.02924 | 0.00077 | 12.333 | 12.33 | 12.333 | 12.33 | 12.33 |
| Cultivars | 3 | 0.72547^*^ | 0.53265^*^ | 0.64507^*^ | 0.27678^*^ | 402.962^*^ | 218.121^*^ | 711.763^*^ | 828.04^*^ | 11.2048^*^ |
| AA | 2 | 1.45087 ^*^ | 0.25760 ^*^ | 0.66903 ^*^ | 0.00561^ns^ | 11.595 ^*^ | 15.807 ^*^ | 121.846 ^*^ | 60.560 ^*^ | 4.0461^*^ |
| AA × Cultivars | 6 | 0.89764^*^ | 0.23020^*^ | 0.59759^*^ | 0.23702^*^ | 4.952^*^ | 0.670^*^ | 9.027^*^ | 0.981^*^ | 0.4929^*^ |
| Total error | 58 | 0.08723 | 0.04016 | 0.06749 | 0.01786 | 9.271 | 0.980 | 10.567 | 33.788 | 0.7366 |
| CV |  | 33.46 | 30.16 | 28.60 | 33.56 | 14.41 | 8.68 | 15.43 | 17.06 | 27.51 |

ns: not significant, ** significant at 1% and * significant at 5%.

**Table S4.** Analysis of variance of AA and cultivars on some phenolic acids parameters of cabbages.

| Source | df | Gallic acid | Chlorogenic acid | Vanillic | Caffeic acid | Rutin | P-Coumaric acid | Catechin | Ferulic | Benzoic |
| --- | --- | --- | --- | --- | --- | --- | --- | --- | --- | --- |
| Block | 2 | 5.523572 | 5.574702 | 5.499783 | 37.95219 | 37.94429 | 37.95219 | 37.94429 | 37.94429 | 5.497757 |
| Cultivars | 3 | 6.048602^ns^ | 6.271813 ^ns^ | 5.540569 ^ns^ | 805.0766 ^ns^ | 438.0708 ^ns^ | 1418.205 ^ns^ | 1649.074 ^ns^ | 27.23828 ^ns^ | 1184.741 ^ns^ |
| AA | 2 | 5.502487 ^ns^ | 6.319386 ^ns^ | 5.002158 ^ns^ | 28.01302 ^ns^ | 36.376 ^ns^ | 246.9176 ^ns^ | 125.2336 ^ns^ | 13.0246 ^ns^ | 204.554 ^ns^ |
| AA × Cultivars | 6 | 5.448084^*^ | 6.177363^*^ | 5.461626^*^ | 14.82327^*^ | 6.321312^*^ | 22.91423^*^ | 6.938806^*^ | 5.969678^*^ | 17.09762^*^ |
| Total error | 58 | 5.0708 | 5.1250 | 5.0265 | 23.3987 | 6.9368 | 25.9719 | 72.0775 | 6.4535 | 19.6564 |
| CV |  | 35.13 | 31.71 | 30.09 | 35.23 | 15.37 | 9.42 | 16.42 | 18.12 | 28.96 |

ns: not significant, ** significant at 1% and * significant at 5%.

**Table S5.** Analysis of variance of AA and cultivars on some phenolic acids and flavonoid parameters of cabbages.

| Source | df | Acacetin | Pyrogallol | Genistein | Cinnamic acid | Luteolin | Quercetin | kaempferol | Myricetin |
| --- | --- | --- | --- | --- | --- | --- | --- | --- | --- |
| Block | 2 | 0.794232 | 0.813662 | 0.785192 | 13.11742 | 13.11742 | 13.11442 | 13.11442 | 0.784422 |
| Cultivars | 3 | 1.317072 ^ns^ | 1.429492 ^ns^ | 1.061202 ^ns^ | 403.7464 ^ns^ | 712.5474 ^ns^ | 828.8244 ^ns^ | 11.98922 ^ns^ | 594.9637 ^ns^ |
| AA | 2 | 1.042022 ^ns^ | 1.453452 ^ns^ | 0.790032 ^ns^ | 12.37942 ^ns^ | 122.6304 ^ns^ | 61.34442 ^ns^ | 4.830522 ^ns^ | 101.294 ^ns^ |
| AA × Cultivars | 6 | 1.014622^*^ | 1.381922^*^ | 1.021442^*^ | 5.736422^*^ | 9.811422^*^ | 1.765422^*^ | 1.277322^*^ | 6.881897^*^ |
| Total error | 58 | 0.8246 | 0.8519 | 0.8023 | 10.0554 | 11.3514 | 34.5724 | 1.5210 | 8.1706 |
| CV |  | 34.24 | 30.94 | 29.38 | 34.34 | 9.46 | 16.21 | 17.84 | 28.29 |

ns: not significant, ** significant at 1% and * significant at 5%.

**Table S6.** Analysis of variance of AA and cultivars on different total AAs of cabbages.

| Source | df | Total sulfur AA | Total aromatic AA | Total essential AA | Total non-essential AA | Total AAs |
| --- | --- | --- | --- | --- | --- | --- |
| Block | 2 | 3.875343 | 3.954492 | 3.838518 | 54.07444 | 54.06222 |
| Cultivars | 3 | 4.688084 ^ns^ | 5.033612 ^ns^ | 3.901656 ^ns^ | 1241.574 ^ns^ | 673.453 ^ns^ |
| AA | 2 | 3.842705 ^ns^ | 5.107254 ^ns^ | 3.068202 ^ns^ | 38.68874 ^ns^ | 51.63452 ^ns^ |
| AA × Cultivars | 6 | 3.75849^*^ | 4.887403^*^ | 3.779451^*^ | 18.27116^*^ | 5.110236^*^ |
| Total error | 58 | 3.17 | 3.26 | 3.11 | 31.55 | 6.06 |
| CV |  | 14.80 | 9.07 | 15.82 | 17.45 | 27.90 |

ns: not significant, ** significant at 1% and * significant at 5%.


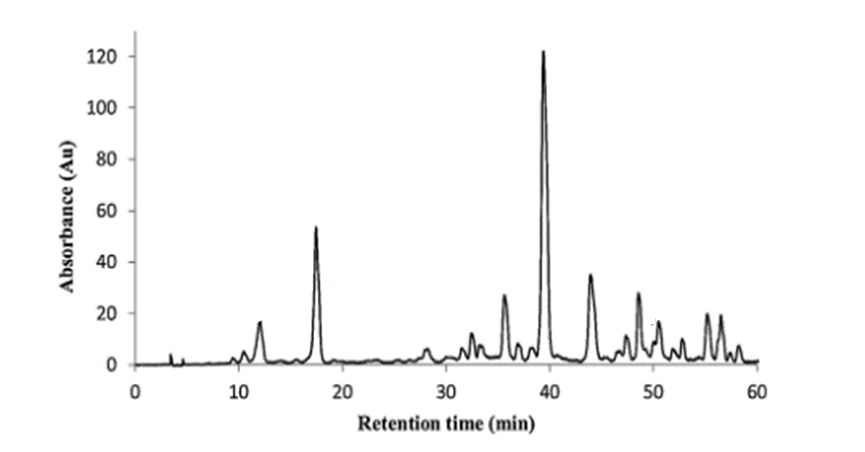


1

2

3

4

5

6

7

8

10

11

13

14

12

9

**Figure S1.** HPLC chromatographic of collard phenolic compound peaks: Gallic acid (1), Caffeic acid (2), Chlorogenic acid (3), P-Coumaric acid (4), Rutin (5), Ferulic (6), Vanillic (7), Cinnamic acid (8), Benzoic (9), Pyrogallol (10), Genistein (11), Luteolin (12), Acacetin (13), Catechin (14).
